# Supplementary material for: The ammonite septum is not an adaptation to deep water: re-evaluating a centuries-old idea
Source: Proc Biol Sci. 2020 Oct 14;287(1936):20201919. doi: 10.1098/rspb.2020.1919 (PMC7657852; doi:10.1098/rspb.2020.1919)
Supplement: Table S4 [file rspb20201919supp4.docx]

| Shell Models | Strain energy (mJ) |
| --- | --- |
| 0.5 mm thick shell |  |
| Trilobate | 10.48 |
| Quadrilobate | 10.32 |
| Quinquelobate | 10.23 |
| Sexilobate | 10.25 |
| Sexilobate models |  |
| 10 mm septal spacing | 11.56 |
| 20 mm septal spacing | 12.23 |
| 0.1 mm thick shell |  |
| Trilobate | 52.66 |
| Sexilobate | 51.20 |
| Koch models |  |
| Iteration 1 | 58.85 |
| Iteration 2 | 58.00 |
| Iteration 3 | 57.63 |
