# Supplementary figures and images for: The ammonite septum is not an adaptation to deep water: re-evaluating a centuries-old idea
Source: Proc Biol Sci. 2020 Oct 14;287(1936):20201919. doi: 10.1098/rspb.2020.1919 (PMC7657852; doi:10.1098/rspb.2020.1919)

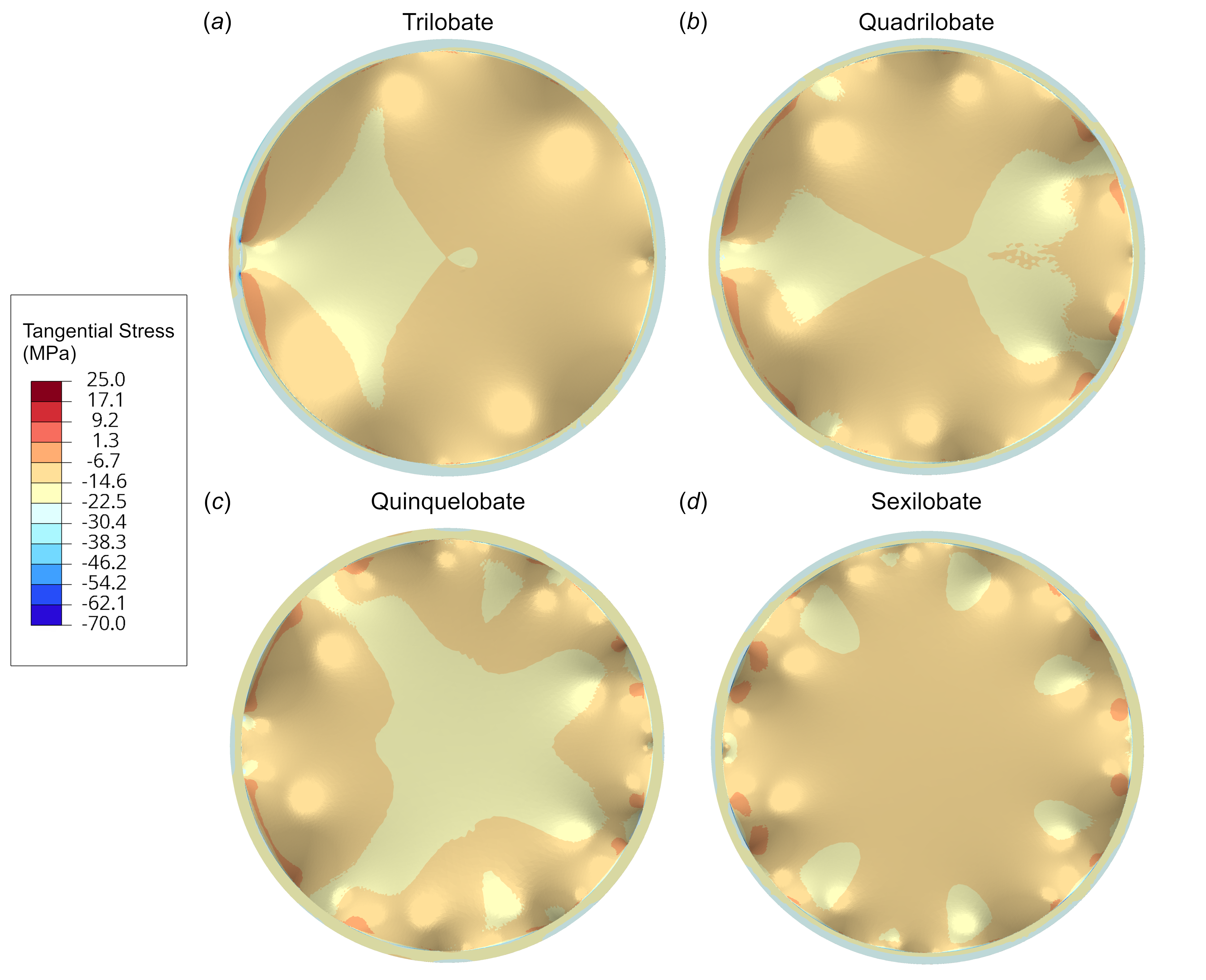

Supplement: Figure S1 [file rspb20201919supp5.tif]
